# Myhre Syndrome Associated With Dunbar Syndrome and Urinary Tract Abnormalities: A Case Report

**DOI:** 10.3389/fped.2020.00072

**Published:** 2020-02-27

**Authors:** Zofia Varenyiova, Gabriela Hrckova, Denisa Ilencikova, Ludmila Podracka

**Affiliations:** Department of Paediatrics, Medical School, Comenius University and National Institute of Children′s Diseases, Bratislava, Slovakia

**Keywords:** Myhre syndrome, Dunbar syndrome, urinary tract defects, renovascular hypertension, TGF-β/BMP signaling cascade, *SMAD4*

## Abstract

Myhre syndrome is a rare condition caused by a mutation in the *SMAD4* gene, which leads to a defective TGF-β/BMP signaling, resulting in the proliferation of abnormal fibrous tissues. Clinically, patients with Myhre syndrome manifest with defects of connective tissue (skin, muscles, joints), and cardiovascular and neurological impairment. In our report, we present a case of a 16-year-old female with skeletal abnormalities, reduced articular mobility, skin, and muscular hypertrophy and cardiovascular defects characteristic of Myhre syndrome. Long-term pulmonary hypertension and arterial hypertension were persistent in spite of antihypertensive treatment. Our patient was also diagnosed with chronic kidney disease and Dunbar syndrome, which is an external compression of the coeliac trunk or coeliac artery by the surrounding tissues. Until now, only a few cases of renal complications in Myhre syndrome have been published. We describe for the first time a female patient with genetically confirmed Myhre syndrome caused by the p.Ile500Val *SMAD4* mutation presenting with an unusual occurrence of congenital vesicoureteral reflux, proteinuria with a decreased renal function, and a condition recognized as Dunbar syndrome.

## Introduction

Myhre syndrome is a very rare condition previously reported in only approximately 60 individuals globally (1). Dysregulation of extracellular matrix synthesis and development is identified as an underlying pathological mechanism of the disease (2). As a result, facial dysmorphism, connective tissue abnormalities, and multiple organ complications affecting skin, heart, and eye tissues typically occur. We present the case of a newly diagnosed 16-year-old female patient with a typical clinical picture of Myhre syndrome combined with rarely seen renal abnormalities and a distinct vascular condition: Dunbar syndrome.

## Case Presentation

A 16-year-old female with noticeably short stature came to our hospital with a history of hypertension and persistent proteinuria >2 g/day. She complained of worsening dyspnoea influencing her everyday physical activities, though the onset of reduced endurance and shortness of breath during exercise had been observed from her preschool age.

**Figure 1:**
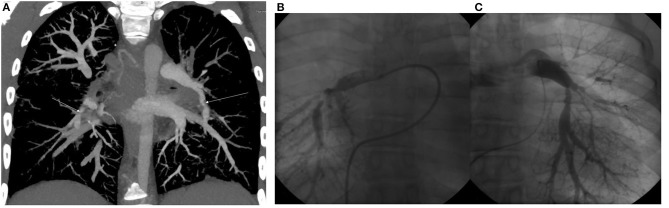
**(A)** CT angiography. Maximum intensity projection in coronal view. Arrows show severe segmental stenoses in the lower right and left lobes. **(B)** Selective pulmoangiography of the right pulmonary artery. We can see severe segmental stenoses of all right lower lobe segments. **(C)** Selective pulmoangiography of left pulmonary stenoses. All segments with moderate to severe stenoses. Decreased arborization pattern of left pulmonary vascular bed.

The patient was the second child of her non-consanguineous Caucasian parents. Her birth weight was 2,490 g and her birth length was 43 cm (both below the 3rd per percentile). In her personal history, a hypoplastic right kidney and bilateral vesicoureteral reflux were detected at the age of six, for which the patient underwent bilateral ureteroplasty. The vesicoureteral reflux was a primary reflux of grade III on the right side and grade II on the left side, with no clinical signs of lower urinary tract dysfunction and a normal uroflowmetry test. During general anesthesia, the patient decompensated with a progression into acute apnoeic event and respiratory failure. The anesthesia was inhalational, and the patient was administered Sevoflurane combined with boluses of opioids (Sufentanil). She was subsequently examined by a cardiologist and diagnosed with 2nd grade mitral valve insufficiency and pulmonary hypertension based on multiple stenoses of the pulmonary artery and its branches (Figure 1). The progression of pulmonary hypertension at the age of nine and the worsening of her overall clinical status with pericardial effusion and dilation of the right ventricle required surgical intervention. However, the dilation of the stenotic branches of the pulmonary artery was only partially successful, and afterwards the patient was recommended ongoing conservative treatment only.

Meanwhile, she had been diagnosed with precocious puberty with menarche at 9 years of age. She was further found to have hearing and visual impairment. Her mental status examined at 14 years old exhibited average mental and intellectual performance with an IQ of 78 (Wechsler Intelligence Scale for Children (WISC III).

When she was admitted, the patient's weight was 60 kg (50–75th percentile) and her height was 140 cm (below 3rd percentile), while the height of her parents was within the standard limits—her mother's height was 170 cm and her father's height was 169 cm.

During her physical examination, the patient showed facial dysmorphism and skeletal changes. Her facial features included an elongated face, a high forehead, a long nose tip, and prominent lips with a smaller mouth and a short, wide neck. Conical teeth, wide interdental spaces and a high arched palate were also present. She had a disproportional figure with a wide, elongated trunk, scoliosis, short extremities, and limited mobility of the elbow and knee joints. Smaller palms and brachydactyly and clinodactyly of the 5th finger on both hands were present. Her feet were wide with a shortened second toe covering normal-length third toes bilaterally. Peripheral non-pitting oedemas were more prominent on the left lower limb; markedly dry skin was observed during physical examination of the patient, with acral predominance of the latter.

Following admission to our hospital, computed angiography, renal function test, renal ultrasound, a DMSA scan, 24-h blood pressure monitoring, skeletal imaging, ophthalmological and otorhinolaryngologic examination, anthropometry, and genetic counseling were indicated in the diagnostic process of her vascular, renal, and skeletal abnormalities.

## Diagnostic Investigation

**Figure 2:**
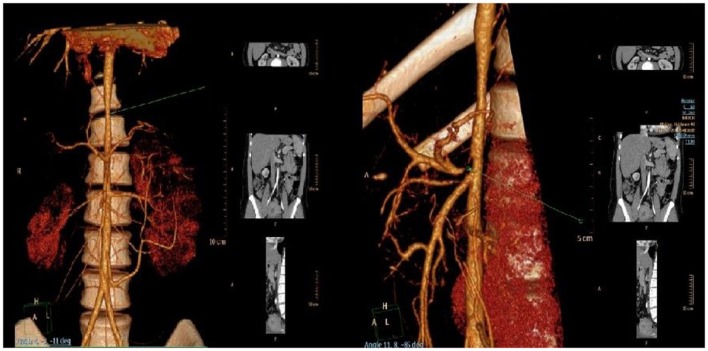
Narrowing of the upper part of the abdominal aorta **(left)** and critical stenosis of coeliac trunk **(right)** visualized by CT angiography.

Computer angiography confirmed the narrowing of the upper part of the abdominal aorta together with a critical stenosis of the coeliac trunk (Figure 2). External compression of the coeliac trunk with the diaphragmatic ligament was identified as a cause of the latter narrowing, something that is known as Dunbar syndrome. Stenoses of renal arteries were not detected. On a renal Doppler ultrasound, the smaller lumen of the right renal artery and a hypoplastic right kidney were present. Based on significant proteinuria (>2 g/day) and impaired renal function [estimated glomerular filtration rate 78.61 ml/min/1.73 m^3^ (3), which is the G2A3 stage of chronic kidney disease according to KDIGO], the patient was diagnosed with reflux nephropathy and reduced function of the right kidney (25%) confirmed by a DMSA scan. Twenty-four-hour blood pressure monitoring revealed systolic hypertension with an average systolic blood pressure of 155 mmHg (>99th percentile based on the patient's height and age) without nocturnal dipping, despite the fact that the patient was regularly administered a combination of antihypertensive drugs. We propose that both vesicoureteral reflux and systemic arterial hypertension contributed to the progression of chronic kidney disease in our patient.

Ophthalmological examination revealed hypermetropia, astigmatism, retinal angiopathy with papilledema, and the tortuous shape of retinal arteries without retinal hemorrhages. In an otorhinolaryngologic examination, bilateral stenosis of the choanae with hypertrophy of nasal adenoids were detected.

On an X-ray, thickened calvarium, scoliosis, fusion of two cervical vertebrae, spina bifida occulta, and the shortened epiphysis of both femoral bones were present.

The anthropometry at her decimal age of 16.7 years showed a borderline-low head circumference −1.4 SD (52.7 cm, 25–10th percentile), short stature −4.4 SD (140 cm, below 3rd percentile), excessive body weight compared to her height +3.0 SD (60 kg, above 98th percentile). The arm span 135.5 cm was adequate to her height and long trunk, when considering her sitting height-to-body height ratio +4.5 SD (above 98th percentile).

## Genetic Counseling

**Figure 3:**
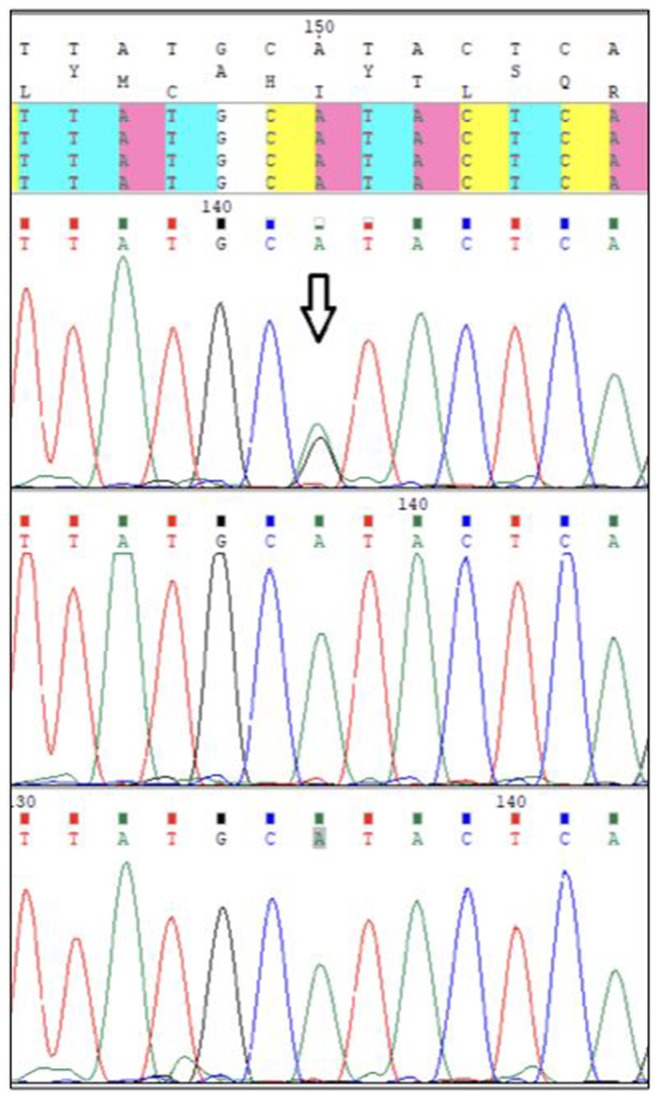
Pathogenic p.Ile500Val *SMAD4* mutation.

The chromosome analysis performed in the first decade of the patient's life confirmed normal female karyotype, i.e., 46, XX. After excluding velocardiofacial syndrome as a phenotype of the 22q11.2 deletion syndrome, the diagnostic process ceased for a couple of years. Multiple diverse anomalies seen during the actual admission led to an extensive differential diagnosis, after which a coexistence of more than one genetic entity in the patient was expected. Short stature, a long trunk, scoliosis and limited mobility of her elbow joints pointed toward the possibility of an inherited skeletal disorder; thus hypochondroplasia (mutations in *FGFR3*) or Leri-Weill dyschondrosteosis (mutations in *SHOX*) were considered (4, 5). Rare cardiovascular abnormalities in the proband were supposed to be caused by mutations of genes involved in the vascular connective tissue development (*ELN* and *SLC2A10*) (6, 7). However, certain clinical features observed in our patient such as short stature, brachydactyly, abnormal muscle build, vision and hearing impairment, together with mal-development of the urinary system, suggested the presence of a different pathology, and a comprehensive literature search drew our attention to Myhre syndrome (mutations in *SMAD4*) (8). Clinical exome sequencing (CES) was indicated to include phenotypically related syndromes with short stature, cardiovascular, urogenital, connective tissue, and other anomalies. The CES revealed the most common *SMAD4* causative mutation (p.Ile500Val) seen in patients with Myhre syndrome (Figure 3) (2). Following a thorough genotype-phenotype correlation in the patient as well as a segregation analysis in her parents, the diagnosis of Myhre syndrome was definitively rendered. The clinical findings in our patient compared to the reported symptoms of Myhre syndrome are summarized in Table 1.

**Table 1:** Clinical picture of the presented patient with Myhre syndrome compared to the reported symptomatology (9, 10).

**Clinical picture of MS**	**Previous cases**	**Presented patient**
Facial dysmorphism	Macrocephaly, hypoplasia of the mid-face, narrowed palpebral fissures, prognathism	Elongated face, conical teeth, wide interdental spaces
Skeletal abnormalities	Short stature, thick calvaria, fused vertebrae, broad ribs, narrow pelvis, brachydactyly	Short stature, thick calvaria, fused cervical vertebrae, spina bifida, scoliosis, brachydactyly, clinodactyly of 5th finger on hands
Limited joint mobility	+	+
Muscular and skin hypertrophy	+	+
Impaired intellectual performance	Behavioral disturbances, intellectual disability, autistic spectrum features	–
Cardiovascular	Persistent ductus arteriosus, aortic or mitral valve stenosis, hypoplasia and/or stenosis of abdominal aorta, pulmonary artery stenosis, pulmonary hypertension, pericardial disorder	Multiple peripheral arterial stenoses, stenoses of pulmonary artery and its branches, pulmonary hypertension, systemic reno-vascular hypertension
Compression of coeliac trunk (Dunbar syndrome)	–	+
Respiratory	Laryngo-tracheomalacia, bronchiolitis obliterans with organizing pneumonia, stenoses and obstruction of upper and lower respiratory system, dyspnea	Bilateral stenosis of choanae, hypertrophy of nasal adenoids, dyspnea
Ophtalmological	Cataract, retinal changes, pseudopapilledema, refractory abnormalities	Hypermetropia, astigmatism, retinal angiopathy, papilledema
Hearing loss	+	+
Impaired pubertal onsent	Precosious or delayed puberty cryptorchidism	Precocious puberty
Urinary	Vesico-ureteral reflux, primary enuresis, hydronephrosis	Bilateral vesico-ureteral reflux, hypoplastic kidney, reflux nephropathy

## Molecular Analysis

Written informed consent from the patient and her parents was obtained prior to the analysis. Genomic DNA was extracted from peripheral blood leukocytes using standard techniques. The DNA libraries were prepared according to CES kit manufacturer's instructions (Clinical Exome Solution, Sophia Genetics) and analyzing their quality control using a DNA 1,000 kit (Agilent). The next-generation-sequencing procedure ran on the NextSeq (Illumina). Genetic variants were analyzed using Sophia DDM software and databases of VarSome, ClinVar, OMIM, and Orphanet. The *SMAD4* causative variant (p.Ile500Val) was confirmed in the patient and excluded in her parents by Sanger sequencing.

## Discussion

Myhre syndrome (MS) was first described in 1981 as a rare condition defined by a combination of the following symptoms: short stature, limited joint mobility, facial and skeletal dysmorphism, muscular hypertrophy, skin anomalies (thick skin), variable intellectual performance together with ophthalmological, and cardiovascular complications (2, 11). *De novo* heterozygous missense mutations the in *SMAD4* gene have been identified as an underlying mechanism of the syndrome via the disruption of TGF-β/BMP signaling cascade involved in the embryonic development of connective tissue, and the cardiovascular and central nervous system (2, 12). Typical morphological features of MS (including narrow palpebral fissures, a small mouth and ears, prognathism, hypoplasia of the mid-face and brachydactyly) were observed during the physical examination of our patient. Radiological signs related to the syndrome were also found (i.e., thick skull bones, broad ribs, scoliosis, and fused vertebrae).

Other symptoms commonly found in MS are ophthalmological and hearing impairment—cataracts, disordered retina, pseudo-papilledema, and refractory abnormalities such as hypermetropia and astigmatism, together with variably expressed hearing loss (1, 13). The affected individuals often present with delayed psychomotor development, autistic features and variably expressed intellectual disability, although none of these were present in our patient (8, 9).

Lin et al. (1) reported that over 70% of patients with MS included in their study manifested with cardiovascular abnormalities, namely persistent ductus arteriosus, aortic or mitral valve stenosis, hypoplasia and/or stenosis of abdominal aorta, pulmonary artery stenosis, pulmonary hypertension and pericardial disorder. Three of the above-mentioned manifestations (pulmonary hypertension, systemic arterial hypertension, and multiple vascular stenoses) occurred in our patient.

A certain spectrum of MS patients requires heart transplants with the risk of the fatal complications of defective wound-healing and excessive post-surgery fibroproliferation (14). Therefore, other than indispensable life-saving, operations, surgical interventions in MS patients should be avoided. Although our patient underwent an open ureteroplasty due to bilateral vesicoureteral reflux, no complications related to post-operative fibroproliferation occurred, demonstrating the wide phenotypic variability of MS.

Interestingly, our patient developed another condition which might be associated with dysregulated proliferation of fibrous tissues—Dunbar syndrome. Dunbar syndrome, or Medial arcuate ligament syndrome, is defined as a condition in which the medial arcuate ligament (MAL) of the diaphragm externally compresses the coeliac trunk or the coeliac artery (15). Our patient did not complain of symptoms such as chronic abdominal pain, dyspepsia, nausea or anorexia, which are the most common clinical manifestations of Dunbar syndrome (16). However, the CT scan and angiography confirmed the diagnosis and explained the absence of typical clinical symptoms in our patient. Blood flow into the lower body compartment was maintained by a Rio-Branco anastomosis which is identified as an anatomical connection between the superior mesenteric artery and the coeliac trunk via pancreaticoduodenal arcades (17). Therefore, our patient did not require any treatment for Dunbar syndrome, except for regular check-ups by a cardiologist. The causative factors of Dunbar syndrome can be both variations in the MAL and the malposition of the coeliac trunk (18). The external compression of the coeliac trunk by the MAL in this case might possibly be caused by a dysregulation of TGF-β/BMP signaling characteristic of MS. The up-regulation of TGF-β/BMP signaling results in an overproduction of extracellular matrix proteins such as collagen and fibronectin in fibroblasts and subsequent fibrosis, in this case possibly resulting in a hypertrophy of MAL (19). However, there is currently no supporting experimental data that would either confirm or deny this hypothesis. Therefore, Dunbar syndrome in our patient can be understood as an additional finding to MS, and therefore the spectrum of clinical signs that are to be assessed in the diagnostic process as well as in the management of such patients should be expanded.

Unique additional features of MS are some abnormalities of the urinary tract. Burglen et al. (8) were the first to report kidney agenesis in a male patient with MS. Other than morphological abnormalities, vesicoureteral reflux, hydronephrosis and primary enuresis have also been described in a minority of MS patients (10, 20). Interestingly, the progressive impairment of renal functions in our patient was initially the main reason for her admission to our clinic. The development of reflux nephropathy (with proteinuria >2 g/day) in the patient resulted in both the progressive worsening of renal functions and in the exacerbation of systemic arterial hypertension (plasma renin levels of 197 mU/l) with a prospective requirement of nephrectomy. Conversely, we propose that long-lasting uncontrolled systemic arterial hypertension in our patient was a contributing factor in the progression of proteinuria. However, we hypothesize that dysregulated TGF-β/BMP cascade characteristic of MS might be only partially responsible for the progression of chronic kidney disease in our patient. Another contributing mechanism that needs to be considered in renal fibrosis is impaired TGF-β signaling with the over-activation of *SMAD3* downstream proteins, which is reported to be a key factor in renal fibrosis (21, 22). We speculate that the systemic arterial hypertension in this case is syndromic, reno-vascular hypertension with the reflux nephropathy playing an important additional role in its exacerbation. These findings represent an important contribution to the diagnostic evaluation of patients with a suspicion of MS as urinary tract abnormalities broaden the spectrum of the clinical symptoms of this rare condition.

Respiratory difficulties, including laryngo-tracheomalacia, bronchiolitis obliterans with organizing pneumonia, stenoses, and the obstruction of both the upper and lower respiratory tracts (such as hypertrophic adenoids seen in our patient) are common findings in MS and might lead to chronic progressive pulmonary inflammation (20). Extrinsic interventions such as intubation often lead to the exacerbation of the existing stenoses due to defective tissue healing (23).

The clinical picture of MS may further include cryptorchidism in males and precocious or delayed puberty that might develop in both male and female patients and is hypothesized to result from impaired hypothalamus-hypophysis signaling (24). Hormonal dysregulation also occurred in our patient, as she developed precocious puberty at the age of nine.

Although early diagnosis of MS is crucial due to the significant risk of life-threatening complications such as pericarditis or laryngotracheal stenoses, the variable expressivity of symptoms makes this condition challenging to recognize (25). The diagnosis of MS is typically established later in life, as the clinical signs are not fully expressed during early childhood and the severity of the symptoms progresses over time, as seen in our patient (11, 25). Early diagnosis of this condition is essential for possible early intervention. A study by (26) suggests that losartan, an angiotensin II receptor blocker, plays a role in the inhibition of TGF-β signaling and therefore deters excessive deposits of extracellular matrix in fibroblasts from MS patients. However, further research is needed to clarify whether MS patients would benefit from this therapy.

In conclusion, this is a very rare case of a female MS patient with an atypical clinical presentation that includes urinary tract complications and associated external compression of the coeliac trunk, which is described as Dunbar syndrome. To the best of our knowledge, we are the first to describe congenital vesicoureteral reflux leading to substantial renal function impairment and the peculiar co-existence of the Dunbar syndrome in a patient with Myhre syndrome.

## Ethics Statement

Written informed consent was obtained from the individual(s), and minor(s)' legal guardian/next of kin, for the publication of any potentially identifiable images or data included in this article.

## Author Contributions

LP, ZV, DI, and GH performed the differential diagnosis. DI and GH contributed to the syndromic evaluation of the patient. ZV wrote the manuscript supported by LP and DI. LP supervised the work.

### Conflict of Interest

The authors declare that the research was conducted in the absence of any commercial or financial relationships that could be construed as a potential conflict of interest.

## Abbreviations

BMP: Bone morphogenic protein
CES: Clinical exome sequencing
DMSA: Dimercaptosuccinic acid
*ELN*: Elastin
*FGFR3*: Fibroblast growth factor receptor 3
KDIGO: Kidney disease improving global outcomes
SS: short stature
mmHg: millimeter of mercury
mU/l: milliunits per liter
MS: Myhre syndrome
*SHOX*: Short stature homeobox
*SLC2A10*: Solute carrier family 2 member 10
*SMAD3*: SMAD family member 3
*SMAD4*: SMAD family member 4
TGF- β: Transforming growth factor beta.
